# Trends in influenza vaccination coverage rates in Germany over five seasons from 2001 to 2006

**DOI:** 10.1186/1471-2334-7-144

**Published:** 2007-12-10

**Authors:** Majbrit V Holm, Patricia R Blank, Thomas D Szucs

**Affiliations:** 1 European Center of Pharmaceutical Medicine, University of Basel, 4031 Basel, Switzerland; 2 Institute of Social and Preventive Medicine, University of Zurich, 8001 Zurich, Switzerland

## Abstract

**Background:**

To assess influenza vaccination coverage from 2001 to 2006 in Germany, to understand drivers and barriers to vaccination and to identify vaccination intentions for season 2006/07.

**Methods:**

9,990 telephone-based household surveys from age 14 were conducted between 2001 and 2006. Essentially, the same questionnaire was used in all seasons.

**Results:**

The influenza vaccination coverage rate reached 32.5% in 2005/06. In the elderly (≥60 years), the vaccination rate reached 58.9% in 2005/06. In those aged 65 years and older, it was 63.4%. Perceiving influenza as a serious illness was the most frequent reason for getting vaccinated. Thirteen percent of those vaccinated in 2005/06 indicated the threat of avian flu as a reason. The main reason for not getting vaccinated was thinking about it without putting it into practice. The major encouraging factor to vaccination was a recommendation by the family doctor. 49.6% of the respondents intend to get vaccinated against influenza in season 2006/07.

**Conclusion:**

Increasing vaccination rates were observed from 2001 to 2006 in Germany. The threat of avian influenza and the extended reimbursement programs may have contributed to the recent increase.

## Background

The severity of influenza and the efficacy of vaccination are well documented in the medical literature. In addition to providing substantial health benefits, vaccination may also be associated with significant economic benefits, not only among the elderly but also among healthy working adults and children. Currently, the German Standing Commission on Immunization (STIKO) recommends influenza vaccination for persons 60 years or older, persons with a chronic disease, persons with an increased professional risk, and if a larger epidemic disease outbreak or pandemic occurs [1]. Some federal states now offer reimbursement of the vaccination to the entire population [2,3]. Despite ongoing efforts by policy makers, physicians and other health care providers, influenza vaccination rates are rarely sufficient to reduce the enormous disease burden.

The WHO states that the risk of a new pandemic is on its highest level since the last pandemic in 1968 [4]. This situation stresses the importance of high immunisation coverage rates in the population.

Previous papers have reported influenza vaccination coverage rates in Germany based on cross-sectional data analyses [5-9]. We have data available for five consecutive seasons and are therefore able to extend the usual cross-sectional approach and to measure potential correlations and to analyse time trends in a consistent dataset.

This paper aims to analyse influenza vaccination coverage rates and related trends over five vaccination seasons in Germany, with a special focus on high-risk group coverage. The second objective is to understand the determinants for being or not being vaccinated, to describe the drivers and barriers to vaccination and to identify vaccination intentions for season 2006/07. In this context, we examine whether the threat of avian influenza has had an impact on vaccination coverage.

## Methods

This survey is part of an ongoing international assessment of influenza immunisation uptake in Europe (France, Great Britain, Italy, Spain and Germany). During five influenza seasons, 2001/02, 2002/03, 2003/04, 2004/05 and 2005/06 a population-based telephone survey was conducted in December among German households representative of the population. The survey included persons aged 14 years and older. The agreement of the interviewees was asked at the beginning of the call. There was no study intervention and the anonymity of the participants was guaranteed. Therefore, no ethical approval was required. Data from official national sources were used as a basis for quota which allowed ensuring the representativeness of the actual respondents.

Four target groups based on national recommendations were specified [1].

- Individuals aged 60 years or older

- Individuals who suffer from a chronic illness

- Individuals who work in the medical field

- Composite target group (individuals aged 60 years or older or who suffer from a chronic illness or who work in the medical field)

According to the German Standing Commission on Immunization (STIKO), the group of chronic illness sufferers is defined as children, adolescences and adults suffering from chronic diseases of respiratory organs, chronic cardiovascular or liver diseases, as well as nephropathies and diabetes or other metabolic disorders [1].

The survey questions were presented in an earlier publication [9]. In the latest season, 2005/06, questions on influenza pandemics and avian influenza were added.

Sample weights were applied to correct for small deviations from the age and gender quota requested and the annual datasets were pooled. Statistical evaluation used SPSS^® ^version 13 for Windows. Bivariate associations of categorical variables were assessed using the Chi squared test. A Chi squared test for trends was used to assess time trends. In the case of continuous variables, differences of means were tested using one-way ANOVA. For all statistical tests, two-sided p ≤ 0.05 was used as the level of statistical significance. Ninety-five percent confidence intervals (CI) were reported as appropriate. Due to the descriptive nature of this data, no correction for multiple testing was made. Predictor variables with strong associations were considered candidates for multivariable analysis and logistic regression was used to identify independent correlates of the outcome of interest, i.e. vaccination coverage.

## Results

### Demographic data

In total 9 990 persons were interviewed. Table 1 gives an overview of the sample. The samples were composed similarly over the years, and were representative of the population aged 14 years and older, in each year.

**Table 1:** Overview of sample

	**2001/02**	**2002/03**	**2003/04**	**2004/05**	**2005/06**	**Total**
**Total **(N)	1 988	1 990	2 006	1 994	2 012	**9 990**

**Mean age **(years) (95%CI)	46.7 (45.9; 47.5)	47.0 (46.2; 47.8)	46.9 (46.0; 47.7)	47.3 (46.5; 48.1)	47.5 (46.7; 48.3)	**47.1 **(46.7; 47.4)

**Male **(95%CI)	47.7% (45.5%; 49.9%)	47.8% (45.6%; 50.0%)	47.7% (45.5%; 49.9%)	47.8% (45.6%; 50.0%)	47.8% (45.6%; 50.0%)	**47.8% **(46.8%; 48.8%)

**Age ≥ 60 years**^1 ^(95%CI)	28.9% (26.9%; 30.9%)	29.6% (27.6%; 31.6%)	29.6% (27.6%; 31.6%)	30.7% (28.7%; 32.7%)	30.6% (28.6%; 32.6%)	**29.9% **(29.0%; 30.8%)

**Work in the medical field**^2 ^(95%CI)	6.8% (5.6%; 7.8%)	6.4% (5.3%; 7.5%)	6.4% (5.3%; 7.5%)	7.2% (6.1%; 8.3%)	7.3% (6.2%; 8.4%)	**6.8% **(6.3%; 7.3%)

**Chronic illness**^3 ^(95%CI)	NA	NA	23.3% (21.4%; 25.1%)	24.2% (22.3%; 26.1%)	22.8% (20.9%; 24.5%)	**23.4% **(22.3%; 24.5%)

**Target group **^1 ^or ^2 ^or ^3 ^(95%CI)	NA	NA	46.2% (44.0%; 48.4%)	47.4% (45.2%; 49.6%)	47.1% (44.9%; 49.3%)	**46.9% **(45.6%; 48.2%)

NA: Not available

### Vaccination coverage rate

**Figure 1:**
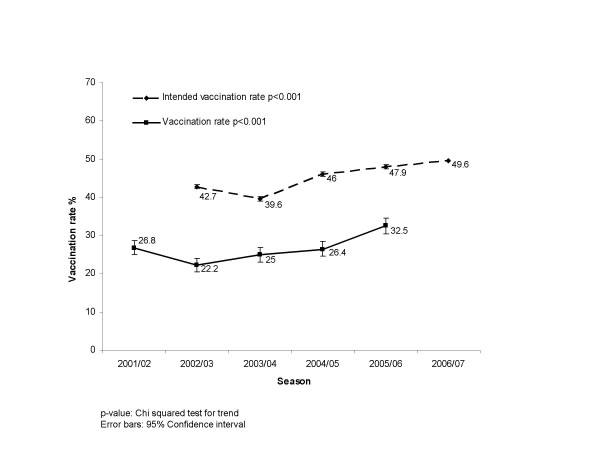
Vaccination rate and intended vaccination rate.

Influenza vaccination coverage reached 32.5% (95% CI: 30.5%; 34.5%) in the latest season 2005/06 (see Figure 1). This uptake was significantly higher than the uptake in season 2004/05 (p < 0.001). The first vaccination coverage rate measured by this series of surveys was 26.8% (95% CI: 24.9%; 28.7%) in season 2001/02. There was a decrease to 22.2% (95% CI: 20.4%; 24.0%) in season 2002/03. Thereafter, the coverage increased to 25.0% (95% CI: 23.1%; 26.9%) in season 2003/04 and to 26.4% (95% CI: 24.4%; 28.3%) in season 2004/05.

With respect to the coming winter of 2006/07, 49.6% of the respondents intend to get vaccinated against influenza (Fig. 1). In season 2005/06 47.9% of the German population intended to get vaccinated.

In the season 2005/06, the proportion of vaccinated persons who had also been vaccinated in the past increased to 29%, the highest rate in this time series (minimum, 20% in season 2002/03; p for trend across seasons <0.001). The proportion of first time immunisations remained essentially stable over time and was 4% in season 2005/06. In contrast, the proportion of persons who had been vaccinated in the past, but not in this season, as well as the proportion of persons who had never been vaccinated before reached minimums of 20% and 48%, respectively, in 2005/06 (p for trend across seasons, 0.131 and 0.002, respectively).

### Vaccination coverage in target groups

**Figure 2:**
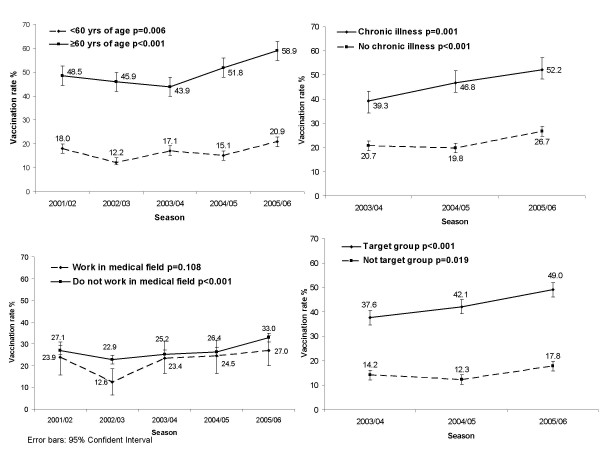
Trend curves of vaccination rates in high-risk groups (p values are for trend over time). **2a**. Age, **2b**. Chronic illness, **2c**. Working in the medical field, **2d**. Combined target group.

The vaccination coverage rate among those above 60 years of age slightly decreased in the beginning of the observation period, but increased since season 2004/05 (see Figure 2a). A positive significant trend was noted over the entire period. In all seasons, the vaccination rate was significantly higher than in persons under 60 years of age (p < 0.001). A question exploring the prevalence of chronic illness was added to the questionnaire in 2003. Over the three observed seasons, significantly higher vaccination coverage rates were observed among the chronically ill compared to the group without chronic diseases (Fig. 2b). Furthermore, the vaccination rate among those with a chronic disease increased over the years. Working in the medical field did not seem to encourage vaccination. The vaccination coverage rate among healthcare workers was lower, but not significantly so, than the coverage rate in the non-medical-professional group (Fig. 2c). The data indicate that the vaccination rate among healthcare workers has been increasing over the last three years, reaching a high of 27.0% in 2005/06. For persons in the combined target group, a significantly higher coverage rate was observed compared to the non-target group respondents (Fig. 2d). For the target group, a significant increase in vaccination rate was observed over the entire period (p < 0.001).

### Influences on vaccination coverage

**Figure 3:**
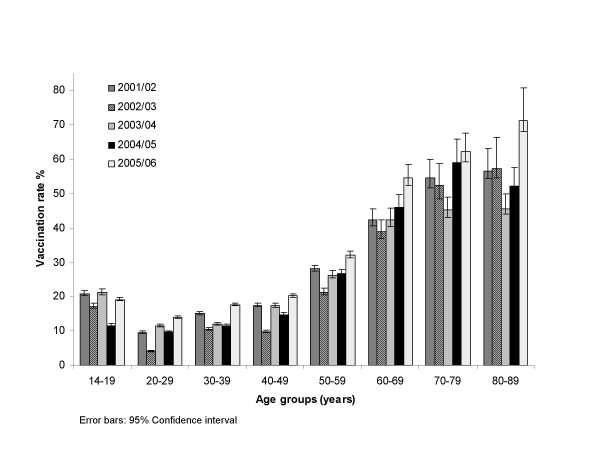
Vaccinated population by age groups.

Vaccination rate differences across age were distinct. Older age and adolescence were associated with higher vaccination rates compared to young adults. In season 2005/06 the uptake was higher for all age groups than in the previous season (see Figure 3).

Pooled data from the first four seasons show a higher vaccination rate among households with few children, a low educational level, and low income. This was confirmed in the 2005/06 data where even further increases were observed. An increase in vaccination rate was also observed among very high-income people, but remained stable for middle size incomes in season 2005/06.

The trend curves illustrated in Figure 2 were not adjusted for potential confounders. The corresponding, unadjusted odds ratios are shown in Table 2. Adjusted odds ratios (not shown) were investigated in the logistic regression models. The adjustment took into account gender, age over 60 years, work in medical field, chronical illness, level of education and income. For years where data on chronic illness, level of education and household income were not available, data were only adjusted for the remaining covariates. The odds ratios for the combined target group were only adjusted for age. In an additional analysis, only persons below the age of 65 years were analysed.

**Table 2:** Likelihood of vaccination coverage in target groups

	**2001/02**	**2002/03**	**2003/04**	**2004/05**	**2005/06**
**Age **(<60*/≥60 yrs)					
OR (95% CI)	4.3 (3.5; 5.3)	6.1 (4.9; 7.7)	3.8 (3.1; 4.7)	6.1 (4.9; 7.5)	5.5 (4.5; 6.8)
p-value	<0.001	<0.001	<0.001	<0.001	<0.001

**Work in the medical field **(yes/no*)					
OR (95% CI)	0.8 (0.6; 1.3)	0.5 (0.3; 0.8)	0.9 (0.6; 1.4)	0.9 (0.6; 1.3)	0.7 (0.5; 1.1)
p-value	0.110	0.026	0.204	0.672	0.151

**Chronic illness **(yes/no*)					
OR (95% CI)	-	-	2.5 (2.0; 3.1)	3.6 (2.9; 4.5)	3.0 (2.4; 3.7)
p-value	-	-	<0.001	<0.001	<0.001

**Combined target group **(age, work in medical field and chronic illness) (yes/no*)					
OR (95% CI)	-	-	3.6 (2.9; 4.5)	5.2 (4.2; 6.5)	4.5 (3.6; 5.5)
p-value	-	-	<0.001	<0.001	<0.001

OR = Odds Ratio (unadjusted). P-value (unadjusted): Pearson Chi squared. * Reference category.

Age 60 or older and chronic illness were independent significant predictors of vaccination (unadjusted OR in 2005/06: 5.5). If only persons below the retirement age (65 years) were included, an increased coverage rate in healthcare workers became visible, and became significant in season 2004/05 (multivariate adjusted OR in 2004/05: 1.5), but no other significant effects were observed for this target group. In the chronically ill, adjusting for age decreased the odds ratio of being vaccinated. These were the only substantial effects of multivariate adjustment, i.e. all other odds ratios remained substantially unchanged. Of the regression covariates not representing target groups, higher household income was significantly associated with a lower vaccination rate. No independent associations with gender or level of education were detected.

### Drivers and barriers for vaccination

Among those who indicated that they had been vaccinated in the current season, the most frequently stated reason was that influenza is considered to be a serious illness and I do not want to get it (see Table 3). Other important reasons for getting vaccinated, not mentioned in Table 3, was older age (38% in 2005/06) and that the social security system pays for it (37% in 2005/06). The attention on avian influenza and on influenza pandemics had influenced the choice of getting vaccinated of 13%. This subgroup was not statistically different from the other vaccinated in terms of age, gender and risk of influenza. However, the proportion of first time vaccinated was higher among those who reported avian influenza as a reason for vaccination (19.3% vs. 10.3%, p = 0.007).

**Table 3:** Ranking of reasons for and against vaccination

	**2001/02**	**2002/03**	**2003/04**	**2004/05**	**2005/06**
**Reason for getting vaccinated – rank (%)**					
Because influenza is a serious illness and I did not want to get it	1 (83)	1 (90)	1 (90)	1 (89)	1 (88)
My family doctor/nurse advised me to do it	2 (66)	2 (72)	3 (70)	2 (73)	2 (68)
So that I do not pass influenza bug to my family and friends	3 (62)	3 (70)	2 (71)	3 (71)	3 (67)

**Reasons for not getting vaccinated (among those never vaccinated) – rank (%)**					
I thought about it, but I did not end up getting vaccinated	-	2 (44)	1 (52)	1 (52)	1 (50)
I do not think I am very likely to catch the flu	1 (38)	1 (47)	2 (41)	2 (41)	2 (44)
It is not a serious enough illness	2 (33)	4 (37)	4 (35)	5 (32)	3 (34)
My family doctor has never recommended it to me	4 (30)	5 (37)	3 (36)	3 (36)	4 (33)
My pharmacist has never recommended it to me	-	3 (38)	5 (32)	6 (32)	5 (31)
I have never considered it before	3 (30)	6(31)	6 (31)	7 (32)	5 (31)

The most common response from those never vaccinated before was thinking about it without putting it into practice (Tab. 3). Not liking needles/injections was commonly reported (31% in 2005/06). Being against vaccination was considered a less important barrier (16% in 2005/06). Persons previously vaccinated but not in the current season (2005/06), said that they did not feel concerned (46%) or they did not think or about getting vaccinated/they forgot (36%).

The knowledge about influenza vaccination was similar across all seasons, despite the increased awareness of pandemic risk in the population. It is well-known that *it is possible to catch influenza even if vaccinated, but that the infection and side effects are then less severe *(see Table 4). Response rates on these questions were high in Germany compared to the other European countries. Twenty-seven percent of the surveyed subjects in Germany agreed with the statement that the influenza vaccine would protect them against avian influenza, although the majority of Germans disagreed with this statement (67%) (Tab. 4).

**Table 4:** Knowledge about influenza and vaccination from 2001 to 2006 (%)

	**Agree**	**Disagree**	**DK**
You can catch influenza even if you are vaccinated against it	77.1	18.5	4.4
If you catch influenza after having had the vaccine, the infection is less severe	65.3	26.0	8.7
The side effects associated with the vaccine (fever, headache, etc.) are acceptable	59.0	28.9	12.1
It is important to get the influenza vaccine each year	56.1	41.8	2.1
The influenza vaccine is not useful if you are in good health	41.6	55.0	3.4
If you have the vaccine you will not catch influenza	34.4	61.7	3.9
The influenza vaccine will protect me in case of avian influenza/influenza pandemic (2005/06)	27.4	67.0	5.6

DK: Don't know

The survey also shows that a recommendation by the family doctor, or knowing more about the efficacy and tolerability of the vaccine or about the disease would encourage many people to get vaccinated. Moreover, reimbursement or a cheaper price of the vaccine might significantly change the level of influenza coverage in Germany.

## Discussion

In Germany, the overall vaccination rate increased significantly in season 2005/06 compared to season 2004/05. The introduction of full reimbursement of vaccination in the entire population by some insurers may have had a direct influence on the increased vaccination rates [3]. Moreover, Germany has been particularly active in terms of media coverage on avian influenza and the possible shortage of antiviral agents. This has increased the population's awareness of pandemic risks and may have influenced vaccination coverage rates. The attention on pandemics and avian influenza may also have encouraged more doctors to recommend vaccination. The actual impact of these potential influences requires further study.

The overall vaccination rate in Europe was 26.5% in season 2005/06. Thus, the vaccination rate in Germany, at 32.5%, was above the European average in this year like in the previous seasons [9]. Our observations on immunisation uptake in the German population are consistent with findings from similar studies performed in Germany [10,6,11].

Vaccination rate differences across age were distinct. Older age and adolescence were associated with higher rates of vaccination. In season 2005/06 the uptake was higher for all age groups (Fig. 3). The increase is very clear in the age group above 60 years, which seems to confirm an effect of the vaccination campaigns. Important increases in the population below the age of 60 may be explained by the extension of reimbursement of influenza vaccination [3].

Another factor affecting vaccination rate is having a chronic disease. Working in the medical field in Germany did not seem to encourage vaccination. Earlier publications on influenza coverage rates note a low coverage in health care workers in Germany [5,12-15]. Leitmeyer and colleagues found a vaccination rate of 22% in season 2001/02 and 26% in season 2003/04 in 20 hospitals [12]. Hallauer and Neuschaefer-Rube found a vaccination rate of 8.4% in season 2001/02 in 25 hospitals [13]. Buchholz and colleagues reported a coverage level of 11.6% among hospital staff in 2000/01 [15]. In comparison, we found a range between 13% and 27% in the population between 2001 and 2006.

Over the last 5 years, most of the vaccinated believed that *influenza is a serious illness they do not want to get*. The non-vaccinated were *thinking about it but never put it into practice*, most likely because they *did not think they were very likely to catch the flu*. Those previously vaccinated but not in the current season (2005/06) said they did not feel concerned, had not thought about it, or simply forgot. The data confirms that the major encouraging factor to vaccination is a recommendation from the family doctor or nurse. This finding was confirmed in other publications [5,8,9,11,16].

Of the 2005/06 respondents, 49.6% intend to get vaccinated in season 2006/07. The gap between *intent to get vaccinated *and actual vaccination was 15–20% over the years. Hence, there is potential to increase vaccination coverage rates in Germany in the future. To achieve vaccine uptake approaching vaccination intention (49.6%), activation of the correct drivers and dealing with the vaccination barriers is to be further implemented in Germany.

Telephone interviews have been used on several occasions to study the vaccination rate in Germany [5,6,8,9,11]. The main advantage of telephone interviews is a potentially high response rate obtained in an affordable and fast manner. The selection process based on random drawing of telephone numbers has been shown to be of high quality [17].

Several restrictions of the present evaluation are recognized. The most important potential reason for selection bias despite correct sampling is non response. However, comparison of face-to-face, mailed and telephone surveys addressing health-related issues, found only small differences between modes of administration and little non-response effects with respect to prevalence estimates [18,19]. Non-response in telephone surveys was found to be less content-oriented than in mailed surveys [20]. Furthermore, bias due to different sociodemographic characteristics of persons not reachable by telephone affected reports of illness and related use of medical services only slightly, provided that the general population was addressed and telephone coverage was at least 90% [20,21]. These published findings support the validity of our approach, although we had no means to independently confirm self-reported vaccination status.

The limitations of the present data collection were described in greater detail in an earlier publication [9]. An increasing problem is the use of wireless telephones. For example, it was shown in the US that people with landlines had a higher odds (1.27) of being vaccinated than those with only access to wireless telephones [22]. If this is believed to be similar in Germany we might have slightly over-estimated the vaccination rate.

## Conclusion

The WHO considers the current influenza pandemic risk to be on its highest level [4]. Efforts should be made at all national and international levels to increase the coverage according to the WHO objectives (i.e. 50% vaccination coverage to be reached in the elderly in 2006 and 75% in 2010) [23]. German has managed to meet the goal as the vaccination rate in the elderly ≥60 of age has reached nearly 60% and 49% on average in all high-risk people. Nonetheless there are still major efforts to be done to reach the 2010 objectives.

## Competing interests

The author(s) declare that they have no competing interests.

## Authors' contributions

MH contributed to the data analysis and wrote the manuscript. PB contributed to the draft and the final version of the manuscript. TS designed the project, contributed to the data acquisition and the analysis and supervised its development.

All authors read and approved the final manuscript.

## Pre-publication history

The pre-publication history for this paper can be accessed here:


